# Abnormal Binocular Rivalry Dynamics in Amblyopia: A Potential Diagnostic Marker

**DOI:** 10.1167/iovs.67.10.66

**Published:** 2026-08-27

**Authors:** Adrien Chopin, Aubrey Rossi, Preeti Verghese, Michael A. Silver, Dennis M. Levi

**Affiliations:** 1 Smith-Kettlewell Eye Research Institute, San Francisco, California, United States; 2 Herbert Wertheim School of Optometry and Vision Science, University of California, Berkeley, California, United States; 3 Sorbonne Université, INSERM, CNRS, Institut de la Vision, Paris, France; 4 Psychology Department, University of Oregon, Eugene, Oregon, United States; 5 Helen Wills Neuroscience Institute, University of California, Berkeley, California, United States

**Keywords:** amblyopia, binocular rivalry, suppression, psychophysics

## Abstract

**Purpose:**

Amblyopia is a neurodevelopmental disorder characterized by monocular deficits and interocular suppression. Binocular rivalry, where perception alternates between dissimilar images, is a powerful tool to probe this suppression. However, the dynamics of sustained binocular rivalry remain poorly characterized in amblyopia. This study aimed to comprehensively characterize these dynamics in individuals with amblyopia compared to visually-normal controls.

**Methods:**

Eleven adults with amblyopia and eight controls continuously reported their perception during a binocular rivalry task through keypresses. We used custom calibration to ensure stable fusion. Key metrics included complete reversal rates (transitions between differing monocular percepts), incomplete reversal rates (transitions to mixed percepts and back), and eye preference indexes.

**Results:**

Although most participants with amblyopia experienced sustained rivalry, their dynamics were severely abnormal. Complete reversal rates were profoundly reduced, and more time was spent seeing the fellow eye percept. Distinct signatures emerged for amblyopia subtypes, shedding light on underlying mechanisms: anisometropic patients exhibited higher incomplete reversal rates than strabismic/mixed patients, suggesting higher binocular noise levels in anisometropic amblyopia. Combining these metrics enabled perfect separation between groups (control, anisometropic, or strabismic/mixed), even with just four minutes of data, in our adult sample.

**Conclusions:**

Binocular rivalry dynamics are fundamentally altered in amblyopia, revealing disrupted reversal mechanisms. These dynamics distinguish amblyopia from normal vision and differentiate between etiologies. The perfect separation achieved suggests that quantifying rivalry dynamics holds significant potential as a rapid noninvasive diagnostic tool.

Interocular suppression is the process in which the visual input from one eye is actively inhibited, or suppressed, in the presence of an input from the other eye. This suppression prevents the brain from perceiving two conflicting monocular images simultaneously (diplopia and confusion). A striking example of interocular suppression is the phenomenon known as binocular rivalry.^1^^,^^2^ Binocular rivalry occurs when two very disparate images (e.g., orthogonally-oriented gratings) are presented simultaneously, one to each eye, at corresponding retinal locations. Although the sensory input is physically stable, the observer's perception typically alternates between the monocular images. This process of perceptual alternation, or reversal, provides critical insights into the interocular suppression mechanisms that operate in the visual cortex.

Amblyopia is defined as a neurodevelopmental monocular visual loss in which the visual acuity of one eye is reduced and cannot be corrected by lenses alone.^3^^,^^4^ Amblyopic deficits include the loss of visual acuity, position acuity, and contrast sensitivity in the amblyopic eye, as well as stereopsis.^5^ These deficits are fundamentally linked to interocular suppression.^6^ Amblyopia is sub-categorized by its presumed etiology: strabismic (caused by a deviated eye), anisometropic (because of different monocular refractive errors), or mixed (a combination of both). Many studies have investigated whether interocular suppression contributes to clinical suppression and binocular rivalry in amblyopia,^7^^–^^13^ or strabismus associated with amblyopia or not.^8^^,^^14^

However, these investigations have often been limited by methodological issues. Many previous studies used brief presentations or flashed rivalrous stimuli, focusing on onset rivalry.^7^^,^^9^^,^^11^^,^^13^^,^^15^^,^^16^ Onset rivalry is now known to exhibit strong, consistent biases in specific visual field locations^17^^–^^19^ and stabilization across brief blank periods,^20^ meaning that it may not reflect the dynamic processes of sustained rivalry observed over extended periods. Furthermore, studies of amblyopia have predominantly focused on the overall dominance of the fellow eye in binocular rivalry as a marker of interocular suppression, rather than the dynamics of the rivalry process,^12^^,^^21^^,^^22^ which can be studied by measuring reversal rates or the time course of perceptual alternations in rivalry.^23^

Crucially, the dynamic properties of rivalry are characterized not only by complete reversals (transition from one monocular percept to the other monocular percept) but also by incomplete reversals (transition to a mixed percept and then back to the preceding percept, also called return transitions). The study of these incomplete reversals is crucial, as they are considered to be a potential marker of binocular noise in the system.^24^ The patterns of complete versus incomplete reversals, and how they differ across subtypes of amblyopia, remain poorly characterized. Moreover, many previous studies did not include separate response options for participants to report a mixed percept or diplopia while experiencing sustained binocular rivalry, potentially contaminating the data with non-rivalrous responses during periods of loss of binocular fusion.

To address these critical limitations and provide a more comprehensive characterization of binocular rivalry in amblyopia, we investigated the dynamics of sustained binocular rivalry in amblyopic patients and controls under conditions optimized to promote binocular fusion (i.e., individual adjustment of interocular contrast differences and ocular alignment). The aims of this study were (1) to confirm that patients with amblyopia can experience sustained binocular rivalry; (2) to compare their rivalry dynamics to those of control participants, specifically focusing on complete and incomplete reversals; (3) to investigate whether these dynamics metrics can differentiate the clinical subtypes of amblyopia (anisometropic vs. strabismic/mixed amblyopia); and (4) to assess the potential of rivalry dynamics as a noninvasive, objective diagnostic tool for amblyopia and its subtypes.

## Methods

### Participants

A total of 19 participants were included in the study. The control group consisted of eight non-amblyopic participants (five female, three male, mean age = 22.6, range 18 to 65) with normal or corrected-to-normal vision, primarily recruited from the University of California, Berkeley. The amblyopic group comprised 11 amblyopic patients (six female, five male, mean age = 44.6, range 22 to 63) recruited through the UC Berkeley Meredith W. Morgan Eye Center. The amblyopic patients were categorized by their etiology: six anisometropic, one strabismic and four mixed amblyopia; given the low number of strabismic and mixed amblyopia patients, we grouped them in one category (strabismic/mixed).

Inclusion criteria for amblyopic patients were a ≥2-line interocular difference in best-corrected visual acuity and the presence of at least one amblyogenic factor (strabismus, anisometropia of ≥1 spherical-equivalent diopters). Exclusion criteria included: use of psychotropic medication, amblyopic eye acuity worse than 20/200 when best corrected, manifest nystagmus, binocular amblyopia, eye deviation ≥30 prism diopters, or a history of diplopia. All participants were kept naïve to the specific purpose of the experiment and provided informed consent prior to participation. One additional amblyopic participant was excluded for being unable to achieve binocular fusion during the experiment. The clinical and visual characteristics of the amblyopic participants are detailed in the Table. This research followed the tenets of the Declaration of Helsinki, was approved by the Institutional Review Board of the Smith-Kettlewell Eye Research Institute, and we collected participants’ informed consent.

**Table.** Visual Characteristics of Amblyopic Participants

	Age/Sex	Type	Amblyopic Eye	Stereoacuity (“)	Refractive Status (Corrected)	Acuity (Best Corrected)	Deviation Angle (PD)	Fusion Time
1	33/F	S	OD	>500	OD: +6.00 DS	OD: 20/100 + 2	4 XT	100%
					OS: +5.50-1.00 × 015	OS: 20/20 + 2		
2	26/F	M	OS	>500	OD: −14.00-1.00 × 020	OD: 20/20-2	8 ET	97.6%
					OS: −12.00-2.00 × 175	OS: 20/40		
3	25/M	A	OS	20	OD: +1.25-1.00 × 002	OD: 20/16	4 EP	100%
					OS: +4.25-1.50 × 170	OS: 20/25-1		
4	54/F	A	OS	50	OD: −0.25-0.75 × 163	OD: 20/20+2	No deviation	97.5%
					OS: +5.25-2.25 × 065	OS: 20/100+2		
5	53/M	A	OS	>500	OD: plano-0.50 × 095	OD: 20/20-1	No deviation	100%
					OS: +4.00-1.50 × 080	OS: 20/50-1		
6	53/F	A	OS	50	OD: +0.50-0.50 × 111	OD: 20/32+1	No deviation	96.7%
					OS: +4.00-1.00 × 030	OS: 20/50-1		
7	22/M	A	OD	40	OD: +2.75-1.00 × 170	OD: 20/50-1	4 XP	96.3%
					OS: +0.25 DS	OS: 20/16+1		
8	63/F	M	OS	50	OD: −3.00 DS (2.5 PD UP)	OD: 20/40-1	4 Hypo	100%
					OS: −2.50 DS (2.5 PD DN)	OS: 20/80-2		
9	63/F	A	OS	>500	OD: +0.75-0.25 × 134	OD: 20/16	6 XP	66.3%
					OS: +5.00-0.50 × 074	OS: 20/80		
10	50/F	M	OS	>500	OD: −0.5 DS	OD: 20/25-1	2 Hypo	97.5%
					OS: +3.00-1.00 × 100	OS: 20/40-2		
11	49/M	M	OS	400	OD: −1.75 to 5.00 × 007	OD: 20/25	14 ET	64.7%
					OS: −3.25 to 2.75 × 002	OS: 20/50-1		

A, anisometropic; DS, spherical diopter; EP, esophoria; ET, esotropia; M, mixed; OD, right eye; OS, left eye; PD, prism diopter; S, strabismic; XP, exophoria; XT, exotropia. Fusion time is the average proportion of a trial time spent before diplopia started.

### Stimuli and Apparatus

**Figure 1.**
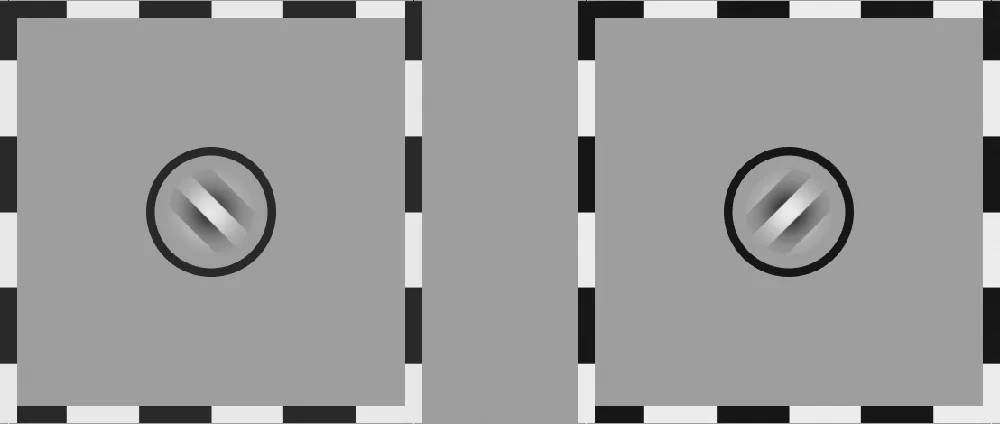
Binocular rivalry stimuli. The left frame and its content were displayed to the left eye, and the right frame and its content were displayed to the right eye, through a four-mirror stereoscope. The central parts (oriented patches) were rivalrous, and the rest of the stimuli were binocular. No adjustments to contrast or vertical/horizontal alignment have been made in this example.

Dichoptic rivalry stimuli consisted of circular oriented grating patches with square wave luminance variation (mean luminance: 40 cd/m^2^) embedded in a 2-D Gaussian envelope (full width at half height: 0.75° of visual angle) limited by an aperture with a diameter of 2° of visual angle (Fig. 1). The spatial frequency of the gratings was 1.5 cycles/°. The stimuli were oriented ±45° from vertical, orthogonally across the two eyes, with orientation randomized across eyes and trials. The weaker eye's contrast was maintained at 0.96 while the other was adjusted through the binocular fusion calibration procedure described below. To promote stable binocular fusion and vergence stability, each rivalrous stimulus was surrounded by a non-rivalrous frame of alternating black-and-white rectangles (width 7°) and a central black annulus (2.25° diameter, Fig. 1).

The stimuli were presented using a custom-made four-mirror stereoscope with a chinrest to stabilize head and eye position. They were generated on a Power Macintosh computer using MATLAB and PsychToolbox^25^ and displayed on a Sony Trinitron Multiscan G500 cathodic monitor (refresh rate 60 Hz) at a distance of 100 cm. The stereoscopic split-screen method was implemented, with the left eye's stimulus presented on the left side of the screen and the right eye's stimulus presented on the right side. The set of stereoscope mirrors directed each stimulus to the appropriate eye. The configuration of the rivalrous and binocular fusion stimuli is illustrated in Figure 1.

### Procedures

#### Binocular Fusion Calibration: The DST

Before the binocular rivalry task, all participants completed a precise individual calibration procedure using the mirror stereoscope to achieve optimal binocular fusion (the Diplopia Suppression Test [DST]).^26^ This involved (1) equalizing accommodation and vergence distances; (2) obtaining vertical and horizontal dichoptic alignment by adjusting the stimulus positions on the screen (sometimes with a 1 DS-lens to correct for any size disparity); and (3) reducing the contrast of the stronger eye's stimulus to match the perceived contrast of the weaker eye's stimulus (including binocular elements). The effectiveness of this calibration was evaluated with two steps:

##### Diplopia Test

Participants reported the number of dots inside three binocular circles (10 trials). If the percentage of “diplopic percepts” (number of perceived dots > number presented) exceeded 20%, calibration was repeated.

##### Suppression Test

Participants gave a “yes/no” response regarding the perceived presence of a dot presented to one eye, inside three binocular circles (15 trials per eye). Calibration was repeated if the sensitivity index (d′) for either eye was less than 1. More information about the DST has been published elsewhere.^26^

#### Binocular Rivalry Task

Participants completed 40 trials of sustained rivalry presentation, each lasting 1 minute, totaling at most 40 minutes of test data for each participant. A trial was stopped if the participant reported an unfused/diplopic percept (using the spacebar). Participants reported their perception continuously using three dedicated keys: left arrow key for the left-oriented grating, right arrow key for the right-oriented grating, and zero key for a mixed percept. Before the 40 trials, the participants completed six practice trials, two of which were catch trials (sequences of binocular presentations mimicking perceptual reversals). Catch trials were used to determine whether the participants understood the task and were accurately reporting their perception.

### Data Analysis

Because of the non-normal distributions in our data, all statistical comparisons were performed using non-parametric Wilcoxon-Mann–Whitney (rank-sum / U) tests. The primary metrics calculated from the continuous perceptual reports were:

1. Complete reversal rate: The rate (events per minute) at which the percept transitioned from one exclusive monocular percept to the other monocular percept (complete reversal), averaged over all trials, after excluding mixed percepts. This rate was defined as the number of complete reversals over all trials divided by the total trial duration in minutes. Note that trials sometimes had different durations, given that some trials were stopped after the onset of diplopia.
2. Incomplete reversal rate: The rate (events per minute) of alternation from an exclusive monocular percept to a mixed percept and then back to the same exclusive percept, averaged over all trials.
3. Dominance of a percept: Proportion of time seeing one particular percept (e.g., mixed percept) over total non-diplopic trial time, calculated for all trials.
4. Eye preference index: Proportion of time in which the stronger eye's percept was experienced over the course of the total exclusive percept time, for all trials. The stronger eye was the eye with the best acuity (fellow eye in amblyopia). An index of 0.5 indicates perceptual balance between the two eyes, calculated as:
  (1) $$\begin{array}{ccc} & & \;\mathrm{Eye}\mathrm{preference}\mathrm{index}\;\\ & & \;\;=\;\frac{Dominance_{stronger\;eye}}{Dominance_{stronger\;eye}\;+\;Dominance_{weaker\;eye}} \end{array}$$
5. Complete reversal index: relative frequency of complete reversals compared to incomplete reversals. A complete reversal index of 1 means that only complete reversals occurred, a value of −1 means that only incomplete reversals occurred), and a value of 0 means that complete reversals occurred as often as incomplete reversals.
  (2) $$\begin{array}{ccc} & & \;\mathrm{Complete}\mathrm{reversal}\mathrm{index}\\ & & \;\;=\frac{\mathrm{complete}\mathrm{reversal}\mathrm{rate}\;\text{--}\;\mathrm{incomplete}\mathrm{reversal}\mathrm{rate}}{\mathrm{complete}\mathrm{reversal}\mathrm{rate}+\mathrm{incomplete}\mathrm{reversal}\mathrm{rate}}\; \end{array}$$
6. Phase duration: duration of a reported percept in seconds.

To assess the semi-automated classification, we use F1-scores, which are the harmonic mean of the positive predictive value (PPV) and sensitivity (SE).

(3) $$\begin{array}{c} F1\;score\;=\frac{2\cdot PPV\cdot SE}{PPV\;+\;SE} \end{array}$$

F1 scores are useful in a diagnostic context in which clinicians usually want to maximize both the proportion of patients correctly diagnosed (sensitivity) and the proportion of true patients in the diagnosed group (positive predictive value). Given the low prevalence of amblyopic patients resulting in unequal proportions of patient/control in our sample, we used the positive predictive value normalized to the prevalence of the patient population. We also reported sensitivity and specificity values, followed by Jeffreys intervals expressed as 95% confidence intervals.

## Results

### Rivalry Dynamics and Group Comparisons

**Figure 2.**
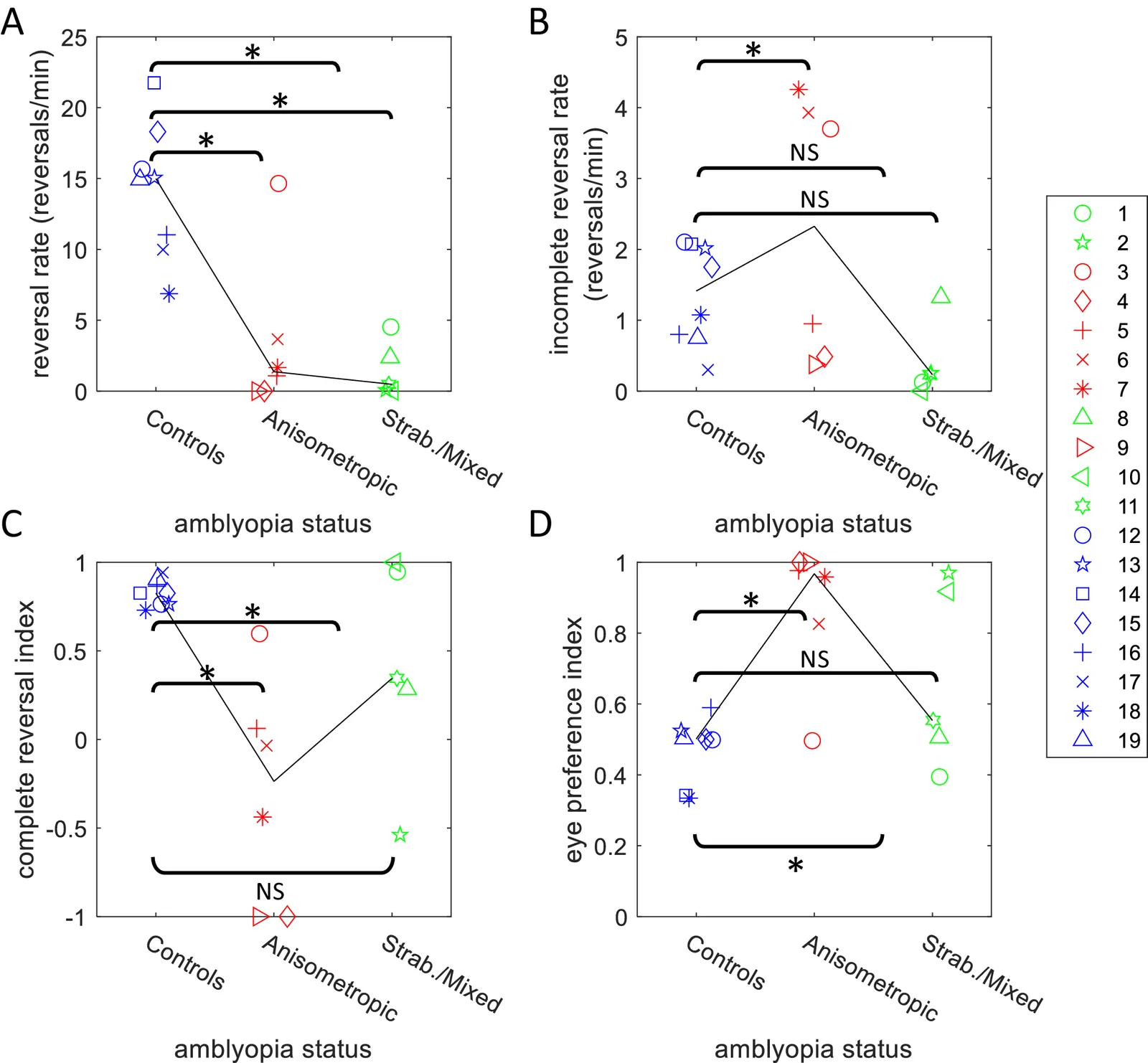
Complete reversal rates **(A)**, incomplete reversal rates **(B)**, complete reversal index **(C)**, and eye preference index **(D)** for each group (*blue*: controls, *red*: anisometropic amblyopic patients, *green*: strabismic and mixed amblyopic patients). Each colored symbol represents an individual subject, showing dispersion around the median (*black line*). *Asterisks* indicate statistically significant differences between controls and either subtype of patients. When the bracket points between the two subtypes, the comparison is between controls and all patients.

All patients reported complete or incomplete reversals (Figs. 2A, 2B). However, all patients showed a combination of decreased reversal rates and increased eye preference (Fig. 2A). Five of the 11 (45%) amblyopic participants reported less than one complete reversal per minute (Fig. 2A). This indicates that the average temporal dynamics of perceptual alternations in amblyopia are severely altered compared to visually normal controls, despite adjusting the contrast in the two eyes for all participants. A detailed time course analysis is presented in the Supplementary Information (Supplementary Fig. S1).

#### Complete Reversal Rate

The rate of complete reversals was significantly lower for the amblyopic group compared to the control group (U = 121, *P* = 0.0002), and this was also the case for each subtype of amblyopia (anisometropic: U = 81, *P* = 0.0047; strabismic/mixed: U = 76, *P* = 0.0016). The control group exhibited a mean of 14.8 ± 4.80 complete reversals/min, and this was drastically reduced in the two amblyopic groups (anisometropic: 3.51 ± 5.48 events/min; strabismic/mixed: 1.45 ± 1.95 events/min; Fig. 2A). This finding indicates a fundamental disruption in the perceptual switching mechanism of binocular rivalry in amblyopia.

Furthermore, complete reversal rate was moderately correlated with amblyopia depth (as measured by the interocular acuity difference at best refraction) when controls were included (Kendall correlation: *r* = −0.58, *P* = 0.0007, large effect size Cohen's *f*^2^ = 1.06). However, the correlation was mostly limited to the anisometropic group (*r* = −0.86, *P* = 0.022, large effect size Cohen's *f*^2^ = 1.13), with no such correlation observed for the entire amblyopic group (*r* = −0.21, *P* = 0.43, small effect size Cohen's *f*^2^ = 0.09). Temporal dynamics analysis (Supplementary Fig. S1) revealed that amblyopic patients maintained a low complete reversal rate across the time course of a trial while controls typically exhibited an initial increase in complete reversal rate immediately after the onset of a trial, followed by a plateau after five to 10 seconds.^23^^,^^27^

#### Incomplete Reversal Rate

Overall, incomplete reversal rates did not differ significantly between amblyopic patients and control participants (U = 91, *P* = 0.39). However, critical differences emerged when analyzing the amblyopia subtypes: anisometropic amblyopic patients exhibited a significantly higher rate of incomplete reversals compared to the strabismic/mixed amblyopia group (U = 48, *P* = 0.03; Fig. 2B), and this was driven primarily by three of the six anisometropic amblyopes. This result suggests greater binocular noise in anisometropic amblyopia^24^ (see Discussion). The complete reversal index (Fig. 2C) was significantly lower for amblyopic than control participants (U = 108, *P* = 0.02), showing that when reversals occur, they tend to be more incomplete in patients than in controls.

#### Eye Preference Index

Unsurprisingly, the eye preference index (quantifying the proportion of time that the stronger eye's stimulus was perceived, excluding mixed percepts), was significantly higher for the amblyopic group compared to controls (U = 49, *P* = 0.009). This index was markedly higher in the anisometropic subgroup, which showed a significant difference from the control group (U = 41, *P* = 0.012; Fig. 2D), whereas the strabismic/mixed subgroup did not differ significantly from the control group (U = 44, *P* = 0.093). This indicates that the anisometropic etiology is associated with pronounced imbalance in rivalry dominance.

#### Diagnostic Marker Potential

**Figure 3.**
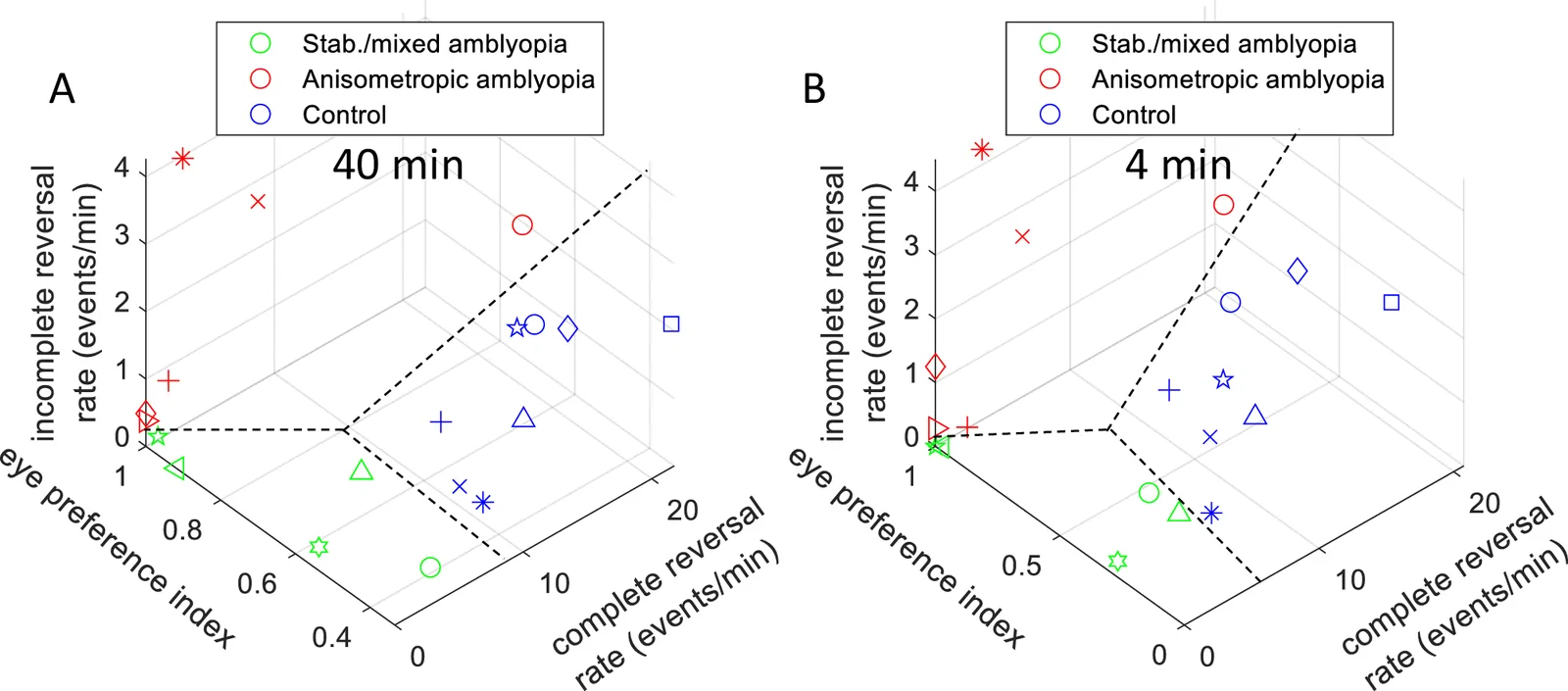
Separation of controls from patients and separation of amblyopia subtypes from one another using a hyperspace consisting of complete reversal rate, incomplete reversal rate, and eye preference index obtained from 40 minutes of data **(A)** or four minutes of data **(B)**. Each colored dot represents an individual subject, with symbols identical to Figure 2, and the color indicates the group (*blue*: controls, *red*: anisometropic amblyopic patients, *green*: strabismic and mixed amblyopic patients). *Dashed lines* show hyperspace boundaries that result in perfect separation of control versus patients and of amblyopia subtype.

Based on the distinct patterns observed in the rivalry metrics, we evaluated a semi-automated classification (with boundaries decided arbitrarily) to assess the diagnostic utility of these metrics (Fig. 3A).

##### Amblyopia Detection Using Only Complete Reversal Rates

The metric of complete reversal rate was highly effective in separating the amblyopia group from the control group, achieving near-perfect separation (specificity: 100% [73.8%–99.99%], positive predictive value: 100%), with only one amblyopic patient falling on the control group side (sensitivity: 90.9% [64.7%–99%], F1-score: 95.2%).

##### Subtype Differentiation Within the Amblyopia Group

Combining the eye preference index and the complete reversal rate measure resulted in complete differentiation of the anisometropic amblyopia patients and the strabismic/mixed amblyopia patients (sensitivity: anisometropia: 100% [67%–99.99%], strabismic/mixed: 100% [62.1%–99.99%]; specificity: anisometropia: 100% [73.8%–99.99%], strabismic/mixed: 100% [73.8–99.99%]; positive predictive value: 100%; F1-score: 100%).

##### Full Classification of Controls and Each Amblyopia Subtype

Adding the incomplete reversal rate measure to the two other rivalry metrics enabled complete separation of all three groups (controls, anisometropic, and strabismic/mixed amblyopia), with all diagnostic metrics at 100% (no error; sensitivity: 100% [80%–100%]; specificity: 100% [73.8%–99.99%], F1-score: 100%). Importantly, this full separation could also be achieved using data collected from only four minutes of sustained rivalry stimulus presentation (the first 30 seconds of the first eight trials), indicating high diagnostic efficiency (Fig. 3B).

Finally, given that our controls and patients with amblyopia differed in age, this is a potential confounding factor. We explored this possibility but found no significant Spearman correlation between age and any of the rivalry metrics (complete reversal rate, controls: *r* = 0.049, *P* = 0.91, patients: *r* = −0.42, *P* = 0.19; eye preference index, controls: *r* = −0.35, *P* = 0.40, patients: *r* = 0.37, *P* = 0.26; incomplete reversal rate, controls: *r* = 0.25, *P* = 0.55, patients: *r* = −0.07, *P* = 0.83; Supplementary Fig. S2).

#### Non-Diplopic Time

Trials were stopped after the participant first pressed the button indicating diplopia. We calculated the average elapsed time in a trial after which observers reported diplopia and expressed it as a proportion of the maximal trial duration. This time in binocular fusion was significantly shorter in amblyopic patients compared to controls (Controls: 99.9% vs. patients: 92.4%, Wilcoxon-Mann–Whitney U = 106, *P* = 0.013).

#### Dominance Proportions

**Figure 4.**
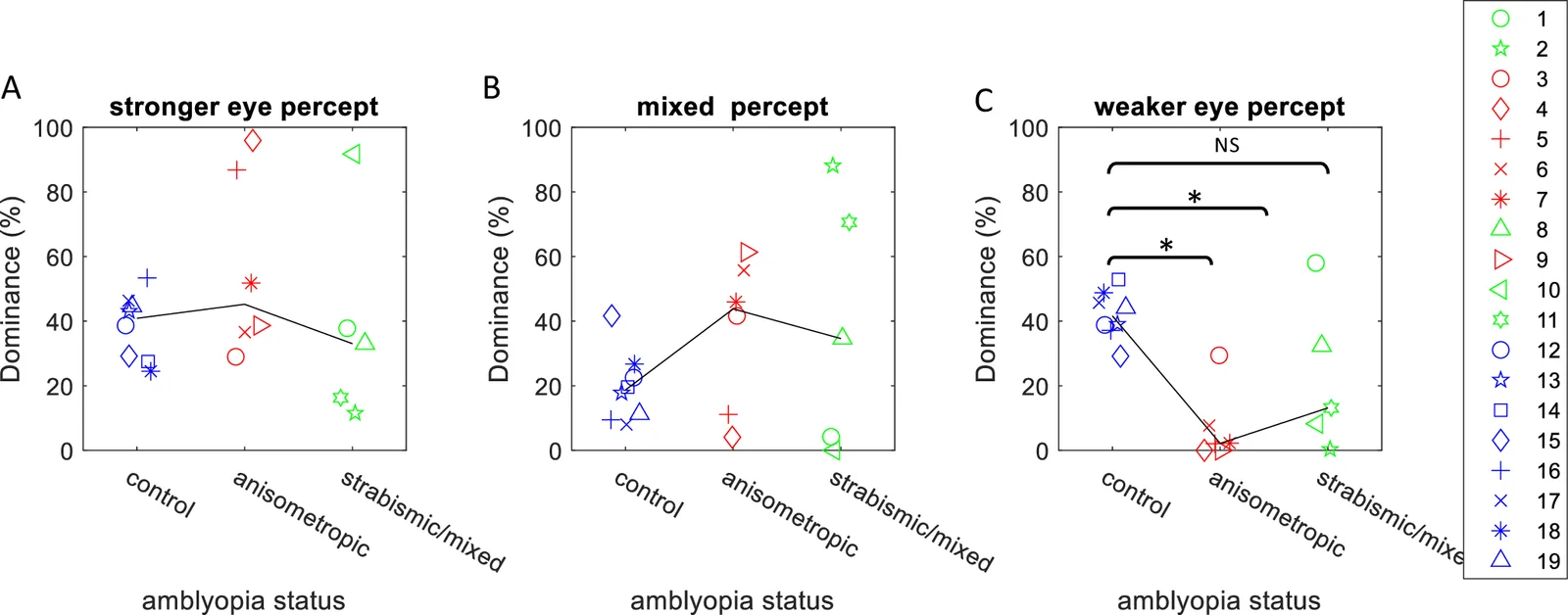
Dominance of the stronger eye percept **(A)**, mixed percept **(B)**, and weaker eye percept **(C)** for each group (*blue*: controls, *red*: anisometropic amblyopic patients, *green*: strabismic and mixed amblyopic patients). Each colored symbol represents an individual subject, showing dispersion around the median (*black line*). *Asterisks* indicate statistically significant differences between controls and either subtype of patients. When the bracket points between the two subtypes, the comparison is between controls and all patients. There was no significant comparison in A and B (tests: controls versus patients, controls versus anisometropic amblyopes, controls versus strabismic/mixed amblyopes).

 Figure 4 shows the proportion of time spent seeing each category of percept. The proportion of trial time where the stronger eye percept was reported was not significantly different between the control and the amblyopic groups (U = 79, *P* = 0.97), although this result may be expected given that the stimulus contrast was often adjusted down in the stronger eye to achieve binocular fusion with the weaker eye. Similarly, there were no significant differences when comparing controls to either the anisometropic (U = 53, *P* = 0.41) or strabismic/mixed (U = 62, *P* = 0.43) subgroups individually.

Conversely, the amblyopic group experienced the weaker eye percept significantly less than controls (U = 114, *P* = 0.0036). This reduction was primarily driven by the anisometropic subgroup, which showed a highly significant decrease compared to controls (U = 83, *P* = 0.0013), whereas the strabismic/mixed subgroup was not significantly different from the control group (U = 67, *P* = 0.13). The dominance of mixed percepts did not significantly differ between the control group and the anisometropic amblyopia group (U = 51, *P* = 0.28) or between the control group and the amblyopic group (U = 68, *P* = 0.35).

##### Phase Durations

**Figure 5.**
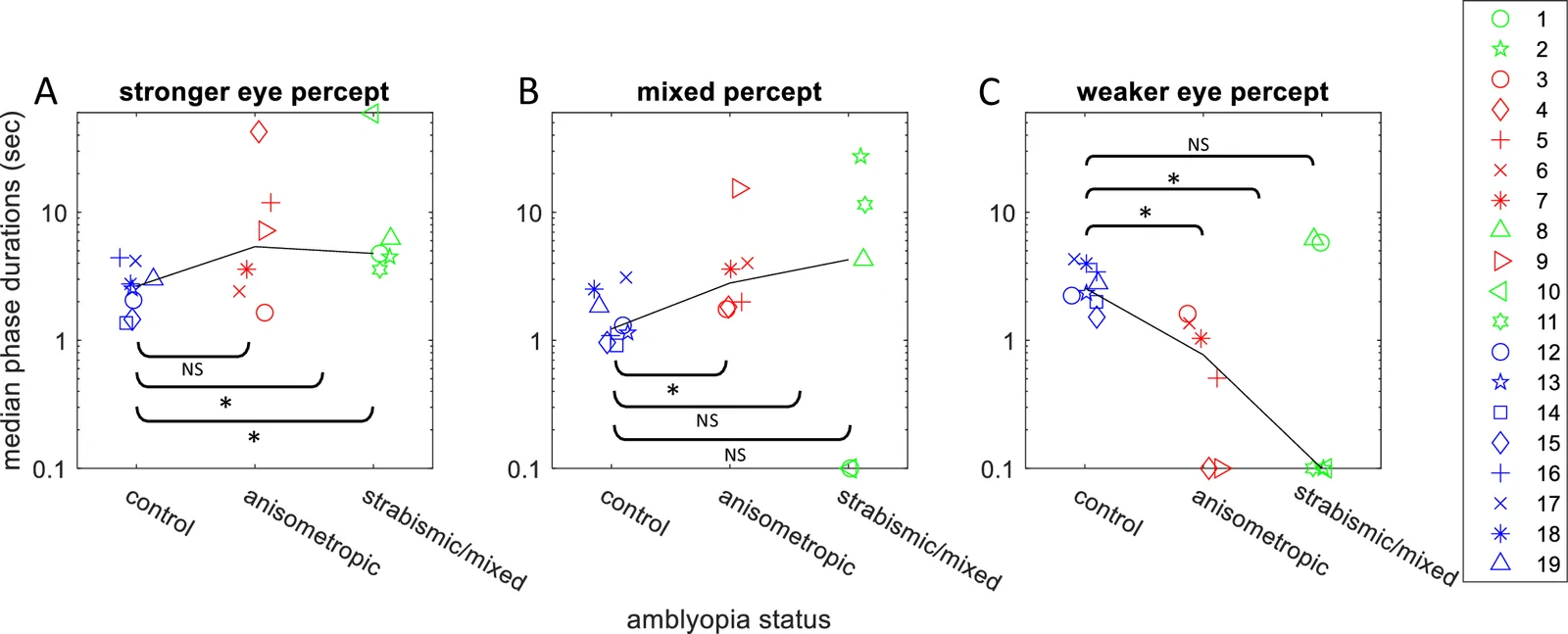
Phase durations for the stronger eye percept **(A)**, mixed percept **(B)**, and weaker eye percept **(C)** for each group (*blue*: controls, *red*: anisometropic amblyopic patients, *green*: strabismic and mixed amblyopic patients). Each colored symbol represents an individual subject, showing dispersion around the median (*black line*). *Asterisks* indicate statistically significant differences between controls and either subtype of patients. When the bracket points between the two subtypes, the comparison is between controls and all patients.

Analysis of average phase durations (Fig. 5) provides crucial insight into the underlying inhibitory mechanisms. The amblyopic group exhibited significantly longer phase durations for the stronger eye percept compared to the control group (U = 50, *P* = 0.012). This effect was driven by the strabismic/mixed subgroup, which demonstrated significantly longer phase durations than controls (U = 38, *P* = 0.0062), while the anisometropic subgroup did not differ significantly from the control group (U = 48, *P* = 0.14).

Furthermore, amblyopic patients exhibited significantly shorter phase durations for the weaker eye percept compared to controls (U = 107, *P* = 0.025). This reduction was highly significant for the anisometropic subgroup compared to controls (U = 83, *P* = 0.0013), but not for the strabismic/mixed subgroup (U = 60, *P* = 0.62). Lastly, median durations of mixed percepts’ phases did not differ significantly between the overall amblyopic group and controls (U = 60, *P* = 0.10) but were significantly longer in anisometropic amblyopia (U = 44, *P* = 0.043).

The observation that phase durations for the weaker eye are significantly reduced in the anisometropic amblyopia group contradicts the modified version of Levelt's proposition II.^31^ This proposition predicts that only the phase durations of the stronger eye would be affected by changes in stimulus contrast/strength, suggesting a fundamentally different binocular disruption mechanism in anisometropic amblyopia. However, the argument is weak because we did not systematically vary the contrast in each eye for each subject.

## Discussion

As a phenomenon that involves interocular suppression, binocular rivalry serves as a promising and intriguing lens for investigating amblyopia. The present study demonstrates that most amblyopic patients experience sustained binocular rivalry but that their average rivalry dynamics are severely abnormal compared to visually normal controls. This strongly supports the hypothesis of abnormal binocular rivalry mechanisms in amblyopia^7^ and appears to contradict previous studies reporting normal rivalry dynamics in amblyopia.^12^

The profound reduction in the rate of complete reversals that we observed, with some patients experiencing none at all despite equating perceived contrast in the two eyes, indicates a fundamental disruption in the reciprocal inhibitory processes that drive perceptual switching between the two eyes’ percepts. Although reversal rates were outside the norm for all patients except one, there is divergence between amblyopia subtypes which allowed us to separate them using their rivalry metrics. Anisometropic amblyopia was mostly characterized by alternations between fellow eye percepts and mixed percepts (resulting in higher eye preference indices, higher incomplete reversal rates, and lower complete reversal indices), while strabismic/mixed amblyopia showed more balanced rivalry (normal eye preference indices, incomplete reversal rates, and complete reversal indices). However, large variability in the strabismic/mixed amblyopia group may have prevented detection of statistically significant differences compared to controls.

### Mechanistic Differences and Subtype Signatures

Our findings reveal distinct mechanistic signatures based on the presumed amblyogenic cause. The significantly lower incidence of complete reversals relative to incomplete reversals (complete reversal index) and the larger incomplete reversal rates in the anisometropic subgroup suggests a greater degree of binocular noise within the system.^24^

**Figure 6.**
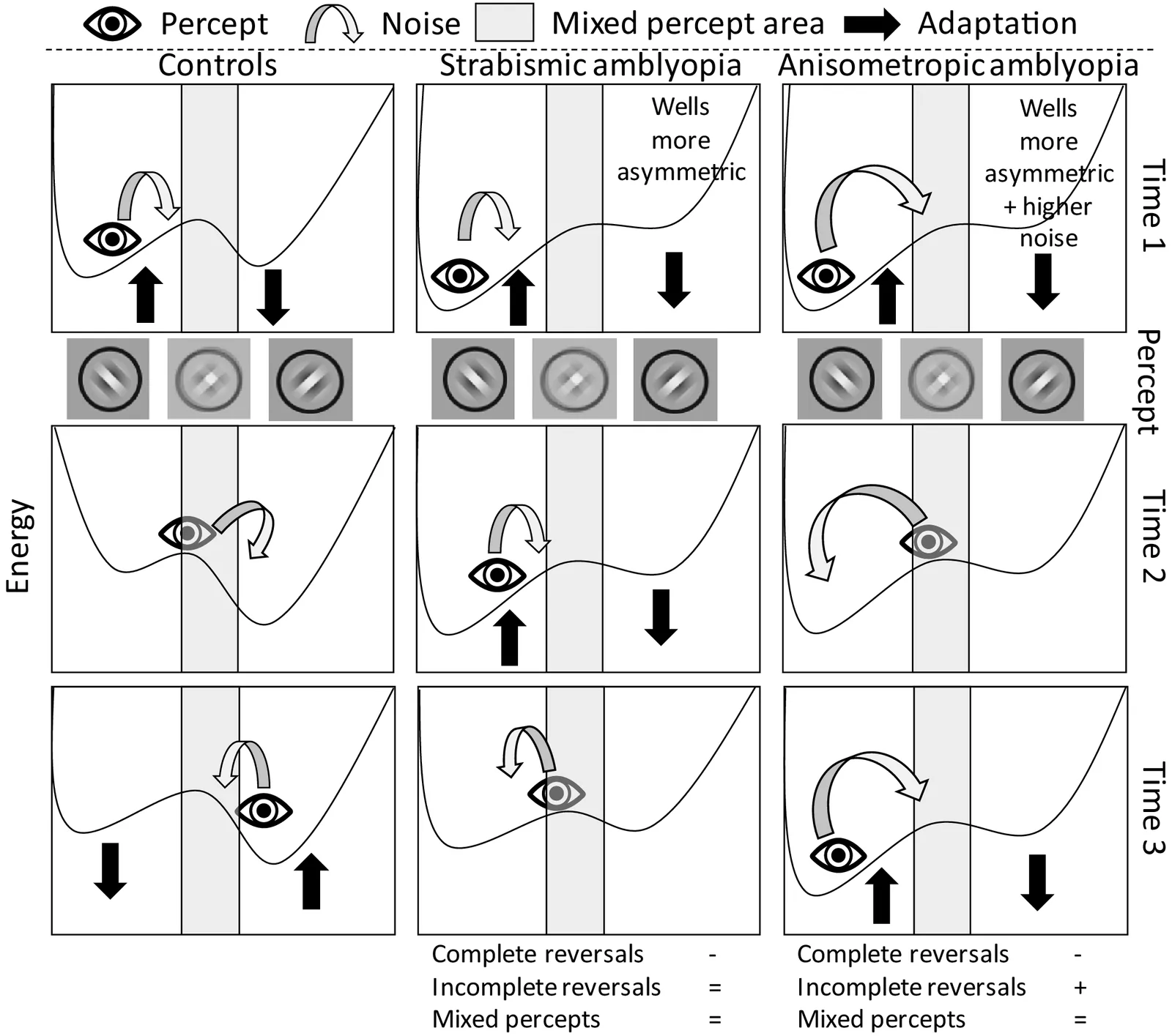
Schematic illustration of a potential double-well framework model. The ordinate shows the energy landscape. The abscissa represents the continuous spectrum of conscious visual perception: the far left represents exclusive dominance of the stronger/fellow eye, the central gray area represents a mixed percept, and the far right represents exclusive dominance of the weaker/amblyopic eye. The eye icon indicates the current perceptual state by its location on the abscissa. The energy landscape is modulated up and down in time through perceptual adaptation. Each panel row corresponds to a different time and each panel column to a different category of subjects (controls, patients with strabismic/mixed amblyopia, patients with anisometropic amblyopia). Binocular noise is indicated by the curved arrow and greater noise is shown with larger arrows (anisometropic amblyopia). Amblyopia is characterized by more asymmetric landscapes with a deeper well for the stronger eye. Although controls experience frequent complete reversals separated by short periods of mixed percepts, and occasional incomplete reversals, patients with strabismic/mixed amblyopia show normal levels of binocular noise that is not strong enough to ”eject” the perceptual state out of the stronger eye energy well (no complete reversal, occasional incomplete reversals with mixed percepts). On the other hand, patients with anisometropic amblyopia have noise levels large enough to frequently push perceptual states into longer mixed percepts, leading to frequent incomplete reversals.

The increased noise hypothesis proposes a potential double-well framework model, with each well corresponding to an exclusive monocular perceptual state (Fig. 6). The transition area between the two wells corresponds to a mixed percept, and the wells’ energy landscape is modulated in time through perceptual adaptation.^28^ In this model, greater noise leads to more frequent incomplete reversals, as we found in anisometropic amblyopia. We also found longer mixed percept phase durations for anisometric amblyopia which predicts that mixed percept dominance should be increased. We did not find such a difference: we speculate that we did not have enough participants to detect a possible increase in mixed percept dominance in anisometropic amblyopia.

The data also indicate that amblyopic participants exhibit skewed perceptual dominance through a reduction of the overall dominance and phase durations of the amblyopic eye and an increase in phase duration of the fellow eye (Figs. 4, 5). However, the patterns differ between amblyopic subtypes with longer fellow eye phase durations observed in strabismic/mixed amblyopia and shorter amblyopic eye phase durations observed in anisometropic amblyopia. These patterns suggest different underlying mechanisms at play in different subtypes of amblyopia and are also compatible with the increased noise hypothesis in anisometropic amblyopia.

### Methodological Considerations

When looking at the temporal dynamics through trial time (Supplementary Fig. S1), we observed that temporal dynamics in the control group were highly comparable to those reported in the literature for sustained binocular rivalry,^23^ validating our stimulus parameters and procedures. Given that lower contrast is associated with lower reversal rates in binocular rivalry,^29^ could the low observed reversal rates in our amblyopia group merely be an artifact of our interocular contrast adjustment procedure for promoting fusion? In that regard, we would actually expect reversal rates to be even lower if we had not used the individual interocular contrast adjustments in the amblyopic group because the goal of this manipulation was to promote binocular fusion and partially lift interocular suppression to allow the best conditions for experiencing binocular rivalry.

Furthermore, we found no significant correlation between the adjusted contrast presented to the fellow eye and complete reversal rate in our patient group (Spearman *r* = 0.03, *P* = 0.94). This suggests that reduced reversal rates are not simply a low-level stimulus contrast effect but rather reflect a deeper central abnormality in the rivalry mechanism.

In addition, the phase duration analysis (Fig. 5) provides further evidence that binocular rivalry dynamics are fundamentally altered in amblyopic patients. If amblyopia were equivalent to reduced stimulus strength in the amblyopic eye and/or increased stimulus strength in the fellow eye, the shorter weaker-eye phase durations partially contradict modified Levelt's proposition II, which states that only dominance durations for the stronger eye should be modulated by changes in stimulus strength. However, it is difficult to hold a firm conclusion here given that interocular contrasts were not systematically manipulated.

A clear limit of our study is that our amblyopic sample size was small (11 participants), and the effects of amblyopia are notoriously inhomogeneous, depending on etiology, age of onset and previous treatment. Although we were able to achieve perfect subgroup separation using the combined three rivalry metrics, we note that the lower bounds of the confidence intervals for this dataset are 80% for sensitivity and 73.8% for specificity, emphasizing the need for replication with a larger sample.

Another limitation of this study is that we only used semi-automated classification (with arbitrary boundaries to separate amblyopia subtypes in the hyperspace of rivalry metrics) which is appropriate only because we have a limited sample. Replication of this work should include a cross-validated classifier to empirically determine these boundaries to allow the boundaries to be used in the parent population without overfitting.

### Relation to Past Studies

It is difficult to compare our results with studies that only focused on binocular rivalry during brief stimulus presentations, as there is insufficient time for perceptual reversals to occur in these studies.^9^^,^^11^^,^^13^^,^^16^^,^^22^^,^^30^ One study reported binocular rivalry in amblyopia but they did not quantify reversals.^13^ Belsunce and Sireteanu^7^ investigated the first six seconds of rivalry trials, but our data show that the first five to ten seconds differ from the rest of a trial, with complete reversals progressively increasing to a plateau (Supplementary Fig. S1).

A very recent study by Hou and Rao^32^ reported a more unbalanced eye dominance and an increased proportion of mixed percepts in adult anisometropic amblyopia during binocular rivalry. We confirm the imbalance in eye dominance and found an increase in mixed percepts in anisometropia but this result did not reach significance in our study. Instead the proportion of incomplete reversals (involving mixed percepts) was increased in individuals with anisometropic amblyopia. Although Hou and Rao's^32^ work aligns with our observation of abnormal binocular integration, they did not quantify the specific rates of complete versus incomplete reversals. By measuring these specific transition dynamics, our study extends their findings to demonstrate how underlying binocular noise signatures can uniquely separate amblyopia subtypes, and patients from control participants.

One study^8^ noted that rivalry occurred in amblyopia, but it focused on pupillary responses and did not measure dominance or reversals. Our finding that binocular rivalry occurs in amblyopia but is severely abnormal both aligns with and contradicts a previous study^12^ that reported typical binocular rivalry dynamics in amblyopic observers on the basis of phase duration distributions. However, there are several major differences between the two studies: we focused on reversal rates; we enrolled adult subjects, including strabismic/mixed amblyopic patients; and we did not exclude participants who reported no perceptual reversals. In contrast, Lunghi studied overall dominance and phase durations only in children with anisometropic amblyopia, and they excluded participants that had no reversals.

Another study with different stimuli also found abnormal rivalry dynamics when looking at dominance measurements but reversal rates were not reported.^21^ Finally, Lawwill and Meur^10^ reported rivalry only for the less severe amblyopic patients (visual acuity better than 20/50 in the amblyopic eye), but they did not quantify reversals.

### Diagnostic Utility and Future Directions

A promising implication of this preliminary study lies in its future diagnostic potential. Amblyopia is the leading cause of monocular vision loss in children. It is a major public health concern affecting approximately 3.3% of the world adult population.^33^ Some clinical measures such as clinical stereotests are poor predictors of amblyopia.^34^ Our classification approach in this adult cohort serves as a critical proof of concept. By jointly using three rivalry metrics to identify amblyopia and separate its subtypes, we obtained perfect separation despite our very limited sample size. Crucially, this separation could be achieved using data collected from only the first four minutes of sustained rivalry presentation, suggesting that these metrics could eventually serve as the basis for a rapid noninvasive screening tool, pending large-scale validation. The lack of consistency of dominance proportions across groups (Fig. 4) further supports using more sensitive metrics such as reversal rates and the eye preference index, rather than raw proportion of time.

Future research should focus on (1) replicating these findings in a larger sample, particularly expanding the strabismic/mixed group to allow the separation of strabismic and mixed subtypes if possible and (2) investigating atypical binocular rivalry dynamics in children, because amblyopia is a developmental disorder best treated early. Because amblyopia is a developmental disorder, the pediatric phenotype may differ significantly from the adult cohort studied here. Specifically, the effects of ongoing visual development, greater neuroplasticity, and differences in prior treatment could result in more variable rivalry dynamics in children compared to the more stable deficits seen in adults. Understanding these developmental differences will be critical for successfully translating these metrics into an early diagnostic screening tool.

Additionally, exploring rivalry dynamics without initial fusion adjustments could further illuminate the usefulness of the rivalry metrics as a diagnostic tool. Finally, using motion rivalry rather than static rivalry would allow the use of optokinetic nystagmus as a proxy for subjective perception during binocular rivalry, thereby opening the doors to an objective diagnostic tool for amblyopia without the need for overt behavioral responses.

## Supplementary Material

Supplement 1
