# Supplementary material for: Abnormal Binocular Rivalry Dynamics in Amblyopia: A Potential Diagnostic Marker
Source: Invest Ophthalmol Vis Sci. 2026 Aug 27;67(10):66. doi: 10.1167/iovs.67.10.66 (PMC13533297; doi:10.1167/iovs.67.10.66)
Supplement: Supplementary file 1 [file iovs-67-10-66_s001.docx]

## Supplementary Information

### Temporal dynamics - time course analysis

To assess the evolution of binocular rivalry over the course of each trial, a time-course analysis was performed on complete reversal rates and perceptual dominance proportions. The instantaneous dominance of the left eye percept at time *t* was calculated as the average proportion of time spent perceiving the left eye percept across all trials on the interval t-1 to t, when data existed for that time point.

Figure S1 illustrates the time course of complete reversal rates averaged across all 40 one-minute trials (Figure S1A), as well as the time course of dominance proportions for the stronger-eye, weaker-eye, and mixed percepts (Figures S1B-D). Consistent with prior reports [^23^](https://www.zotero.org/google-docs/?PHEAME), control participants typically showed an increasing phase followed by a plateau. In contrast, amblyopic participants exhibited low complete reversal rates that were stable over time, indicating that the overall average reversal rate effectively captures the key disruption in their dynamics. A similar stable pattern was observed in all three dominance time courses for the amblyopic individuals.

Figure S1: Temporal dynamics for complete reversal rates (A) and dominance of the stronger eye percept (B), weaker eye percept (C), and mixed percepts (D) for each group (blue: controls, red: anisometropic amblyopic patients, green: strabismic and mixed amblyopic patients). Each colored solid line is an individual subject time course averaged across all available trials for that subject at each time point. The bold colored solid lines depict the median across subjects at each time, and the colored regions indicate standard errors of the mean. Data have been smoothed with a 3-sec moving window.

### Effect of age

Figure S2: Correlation between age and (A) complete reversal rate, (B) eye preference index and (C), incomplete reversal rate for each group (blue: controls, red: patients with amblyopia). Each colored dot is an individual subject. Each panel in the figure includes the correlation r and p values for the controls (in blue) or the patients (in red). None of the correlations are significant.
